# Novel Acetal Rotating Tool Improving Surface Quality in Incremental Sheet Forming

**DOI:** 10.3390/ma19040826

**Published:** 2026-02-23

**Authors:** Hongsong Song, Kai Han, Peng Xu, Jinxin Zang, Xiaoqiang Li, Shaohua Wang

**Affiliations:** 1 AECC Beijing Institute of Aeronautical Materials, Beijing 100095, China; songhongsong@126.com (H.S.); apple_zjx@163.com (J.Z.); 2 Beijing Engineering Research Center of Advanced Aluminum Alloys and Applications, Beijing 100095, China; 3 School of Mechanical Engineering and Automation, Beihang University, Beijing 100083, China; xp14221143@163.com (P.X.); lixiaoqiang@buaa.edu.cn (X.L.)

**Keywords:** incremental sheet forming, surface quality, forming tool, acetal rotating tool

## Abstract

Surface quality remains a critical factor limiting the widespread adoption of incremental sheet forming (ISF). This study proposes a novel acetal wheel-type rotating tool and systematically investigates the influence of tool structure and material on surface quality through experimental and numerical analyses. The results demonstrate that the acetal tool significantly improves surface quality by 32–40% compared to conventional metal ball tools, primarily due to enhanced sheet–tool contact area that effectively suppresses the formation of surface waviness topography. Furthermore, the wheel-type tool reduces contact stress by over 37.5% while improving surface quality by 21.1% relative to traditional metal ball tools. Notably, the acetal rotating wheel achieves a 32.0% surface quality enhancement while eliminating tool wear, highlighting its industrial applicability. These findings provide both a mechanistic understanding and a practical tooling solution, highlighting significant potential for improving surface quality in ISF industrial applications.

## 1. Introduction

The rapidly accelerating product iteration cycles in modern manufacturing necessitate highly efficient and cost-optimized component fabrication solutions during research and development phases. Incremental sheet forming (ISF) has emerged as a transformative technology for thin-walled component production, offering unparalleled advantages in rapid small-batch manufacturing with minimal tooling investment, thereby attracting substantial research interest across the aerospace and automotive industries [1,2]. Nevertheless, broader industrial implementation of ISF continues to face critical challenges stemming from inherent surface quality limitations, most notably the persistent issues of periodic waviness formation and tool-path induced surface defects.

The dominant surface waviness, a macroscopic geometric artifact, primarily governs surface integrity in ISF. Regarding its formation mechanism, Han et al. [3] and Chang et al. [4] attributed this phenomenon to residual deformation effects, providing fundamental understanding of its origin. Subsequent research has developed predictive models through two complementary approaches: Durante et al. [5], Hagan and Jeswiet [6] established surface-roughness prediction based on tool geometry residual height, while Han et al. [3] and Chang et al. [4] further advanced modeling accuracy by incorporating material deformation mechanisms into their theoretical framework for both surface-roughness and waviness prediction. Parameter optimization studies [7,8,9,10,11] have systematically investigated process control methods, revealing that increasing tool diameter and reducing step depth significantly improve surface quality, whereas lubrication conditions and spindle speed exhibit relatively minor influence. However, due to the inherent characteristic of localized rigid tool–material contact in ISF process, such parametric adjustments can only mitigate but not completely eliminate the process-specific waviness patterns.

Recent advances in tooling development have focused on improving surface quality in incremental forming processes [12,13]. Kim and Park [14] and Lu et al. [15] introduced ball rolling tools, while Xu et al. [16] developed laser-grooved tools, both achieving notable surface quality improvements while still retaining some waviness patterns. Him et al. [17] proposed a fixed rubber-ball forming tool that effectively reduced surface waviness, although the underlying mechanism remains unclear and the tool suffers from severe wear during operation, leading to limited service life. Alternative approaches employing backing plates have shown promising results. Skjoedt et al. [18] and Alves et al. [19] demonstrated that single-point incremental forming with backing plates could significantly enhance surface quality and eliminate waviness. Li et al. [20] further revealed that the backing plate increases the contact area between tool and sheet metal while preventing relative friction, thereby suppressing waviness formation. However, the method of backing plate could face limitations in large-scale applications, where high through-thickness stresses cause plate extrusion and subsequent fracture [21].

To address the surface-quality limitations hindering the wider industrial adoption of ISF, this study aims to systematically investigate the effects of tool material (acetal vs. metal) and tool structure (ball vs. wheel-type) on the surface quality, contact mechanics, and geometric accuracy of formed parts. The purpose is to elucidate the underlying mechanisms through integrated experimental and numerical analyses, and to evaluate the potential of a novel acetal rotating-wheel tool for practical applications. By clarifying how material compliance and geometric design jointly influence surface generation, this work seeks to provide a viable tooling solution that can overcome a key barrier in ISF technology.

## 2. Method

### 2.1. Experimental Setup

**Figure 1:**
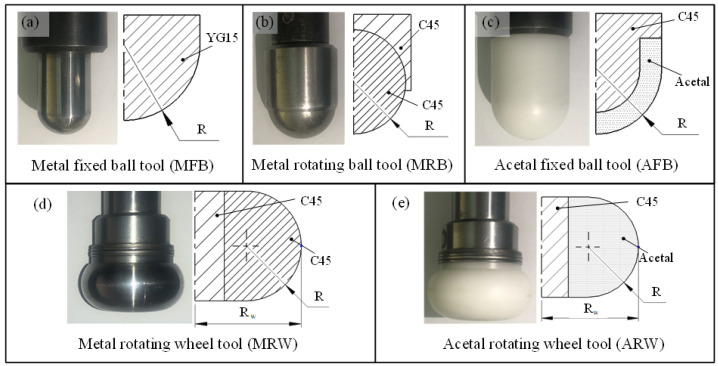
Illustration of different forming tools: (**a**) metal fixed ball tool (MFB); (**b**) metal rotating ball tool (MRB); (**c**) acetal fixed ball tool (AFB); (**d**) metal rotating wheel tool (MRW); (**e**) acetal rotating wheel tool (ARW).

This study developed five forming tools with systematic variations in geometry, material, and motion characteristics (Figure 1). The toolset includes conventional metal fixed ball (MFB) tools using YG15 carbide, metal rotating ball (MRB) tools made of C45 steel enabling free rotation for rolling contact, and acetal fixed ball (AFB) tools featuring polymer contact surfaces. Beyond these standard configurations, we introduced two innovative wheel-type tools: a metal rotating wheel (MRW) and an acetal rotating wheel (ARW), both maintaining the same cross-sectional radius (R) as ball tools while featuring an increased circumferential radius (R_W_). The initial surface roughness of all tools was controlled to be low (Ra < 1.6 μm).

**Figure 2:**
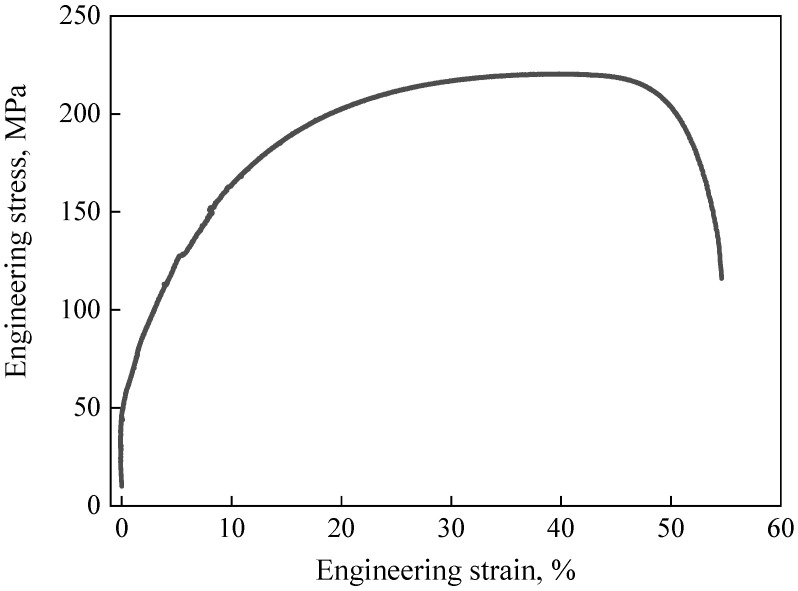
Material flow stress of copper.

**Figure 3:**
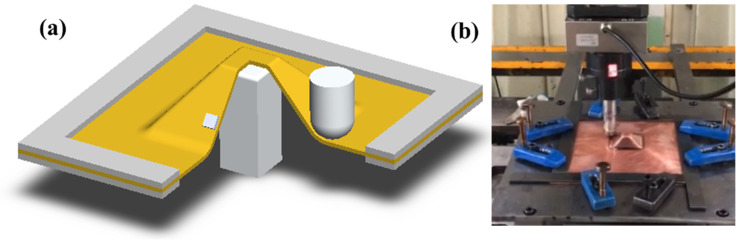
(**a**) ISF schema for a truncated pyramid part and (**b**) ISF process.

The present study employed commercial pure copper (Cu 99.92 wt.%, Fe 0.03 wt.%, and Si 0.05 wt.%) as the research material. The as-received sheet was in an annealed state with an average grain size of approximately 14.7 µm [22]. Its engineering stress and strain curve presented in the accompanying Figure 2. Mechanical characterization revealed that yield strength 50.2 MPa, ultimate tensile strength 221.2 MPa. The specimens were fabricated into 45° square pyramidal shapes using two-point incremental sheet forming process as shown in Figure 3. Key process parameters included a step depth of 1.0 mm, feed rate of 3000 mm/min, and forming tool radius of 10 mm as shown in Table 1. Post-forming characterization involved surface quality and geometric accuracy. Cross-sectional specimens were precisely extracted from the pyramid side walls using wire electrical discharge machining (EDM, DK7732, Jiangsu Dongqing CNC Machine Tool Co., Ltd., Taizhou, China) for subsequent surface topography measurement via confocal laser scanning microscopy (OLS5000, Olympus Corporation, Tokyo, Japan). Process forces during forming were monitored in three orthogonal directions using a multi-component dynamometer (FC3D160, Shanghai Naichuang Testing Technology Co., Ltd., Shanghai, China). Furthermore, the geometric accuracy of formed components was quantitatively assessed through coordinate measuring machine (CMM, HSCAN300, Hangzhou Scantech Technology Co., Ltd., Hangzhou, China) inspection.

### 2.2. Numerical Simulation

**Figure 4:**
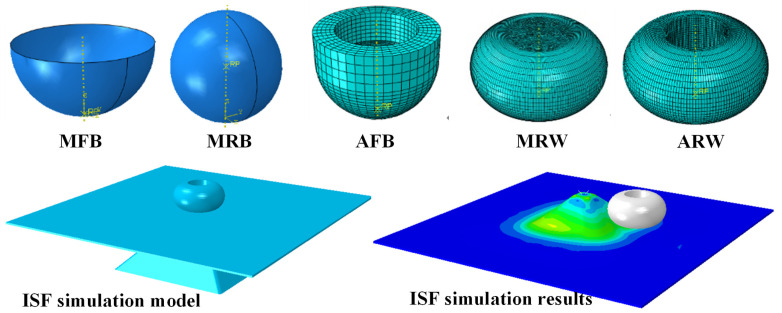
Finite element model of ISF process using different forming tools.

To better elucidate the underlying mechanisms of surface-morphology formation, this study developed finite element models for different forming tools to analyze the evolution of contact stress and strain during the forming process. The commercial software Abaqus/Explicit 6.14 was employed for its capability in handling large deformation and complex contact conditions. The simulation setup mirrored the experimental conditions as shown in Figure 4, employing C3D8R elements for the sheet metal with a uniform mesh size of 1.0 × 1.0 × 1.0 mm^3^.

For the forming tools, distinct modeling approaches were implemented based on material behavior. Metal tools (MFB, MRB, and MRW), exhibiting negligible deformation due to their high strength, were modeled as rigid bodies. The ball MFB and MRB tools were represented as analytical rigid bodies, while the wheel-type MRW tool was discretized as a rigid mesh with 1.0 mm element size. The acetal tools (AFB and ARW), undergoing elastic deformation during forming, were modeled as deformable bodies using C3D8R elements with 1.0 mm mesh size.

The sheet metal was modeled using an elastic-plastic material model. The plasticity data were defined by the engineering stress–strain curve obtained from tensile tests, which was converted into true stress–strain data for the simulation. The acetal tools (AFB and ARW) were modeled as deformable bodies with a linear elastic material model, using a Young’s modulus of 2800 MPa and a Poisson’s ratio of 0.35. The contact between the tool and the sheet was defined using a surface-to-surface contact algorithm with a penalty formulation with a friction coefficient of 0.1. The finite element modeling framework employed in this study has been rigorously validated against experimental data in our prior research [22]. The predictive capability of the finite element model was further validated by comparing the simulated and experimentally measured forming forces for all five tool configurations, as presented in the Supplementary Materials. The results confirm the reliability of the model for comparative contact mechanics analysis.

## 3. Results

### 3.1. Surface Topography

**Figure 5:**
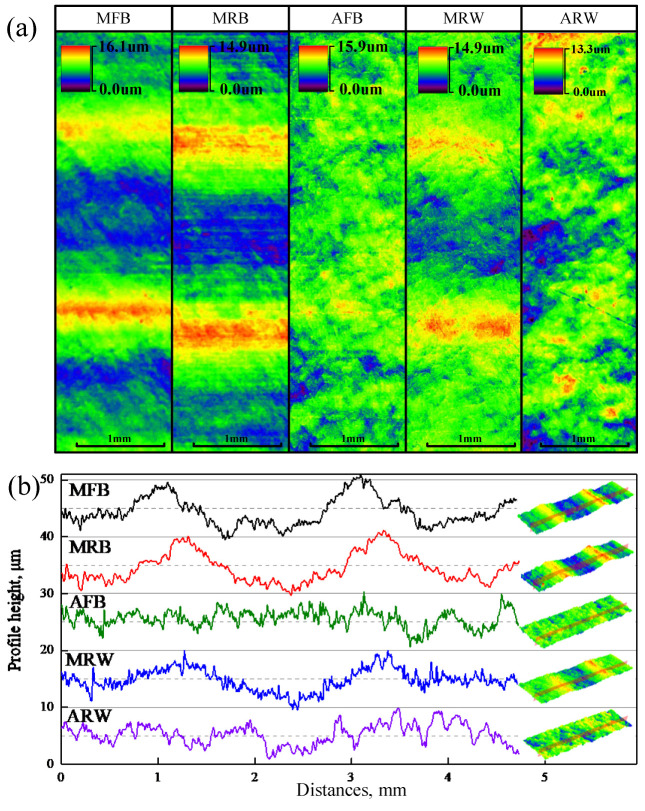
The surface of material contacting different forming tools: (**a**) surface topography; (**b**) surface outline.

The surface morphology of formed parts was characterized using confocal laser microscopy, revealing distinct differences between tool materials. As shown Figure 5a, metallic tools (MRB, MFB, and MRW) produced pronounced periodic peaks and valleys aligned with the forming direction, while acetal tools (AFB and ARW) generated more randomized surface textures. Cross-sectional profiles perpendicular to the forming direction (Figure 5b) confirmed that metallic tools exhibited the characteristic waviness pattern inherent to ISF process, whereas acetal tools effectively suppressed this waviness formation.

**Figure 6:**
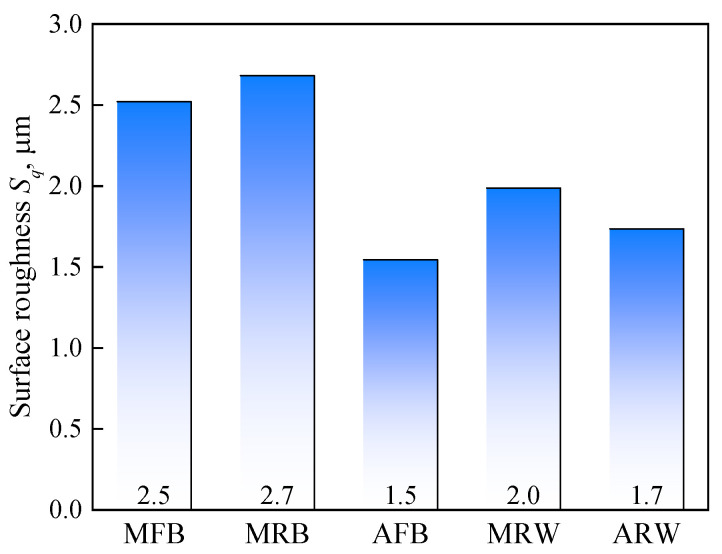
Surface roughness (root mean square height, $S_{q}$) of formed part using various tools.

Quantitative analysis of surface roughness showed that while peak roughness values were similar across tools, the calculated root-mean-square deviation of the surface $S_{q}$ demonstrated significant variations as shown in Figure 6, which is defined as follows:

(1) $$S_{q}\;=\;\sqrt{\frac{1}{A}\iint Z{\left(x,\;y\right)}^{2}dxdy}$$

The conventional metal tool (MFB) yielded an $S_{q}$ of 2.5 μm. In contrast, acetal tools showed superior performance, with AFB producing the smoothest surface with $S_{q}$ of 1.5 μm by 40% decrease and ARW achieving an $S_{q}$ of 1.7 μm by 32% decrease. Among metallic tools, the $S_{q}$ of MRB is 2.7 μm with a slight change compared to MFB, while the wheel-type MRW demonstrated more substantial enhancement, in which $S_{q}$ is 2.0 μm with 21.14% reduction over MFB.

The experimental results clearly demonstrate that acetal tools can effectively eliminate the characteristic waviness in incrementally formed parts while significantly improving surface quality. Additionally, the wheel-type tool design (MRW) shows notable advantages over conventional ball metal tools in surface finish enhancement. Both material selection and tool geometry optimization are crucial factors for surface quality control in ISF processes.

### 3.2. Contacting Area and Stress

**Figure 7:**
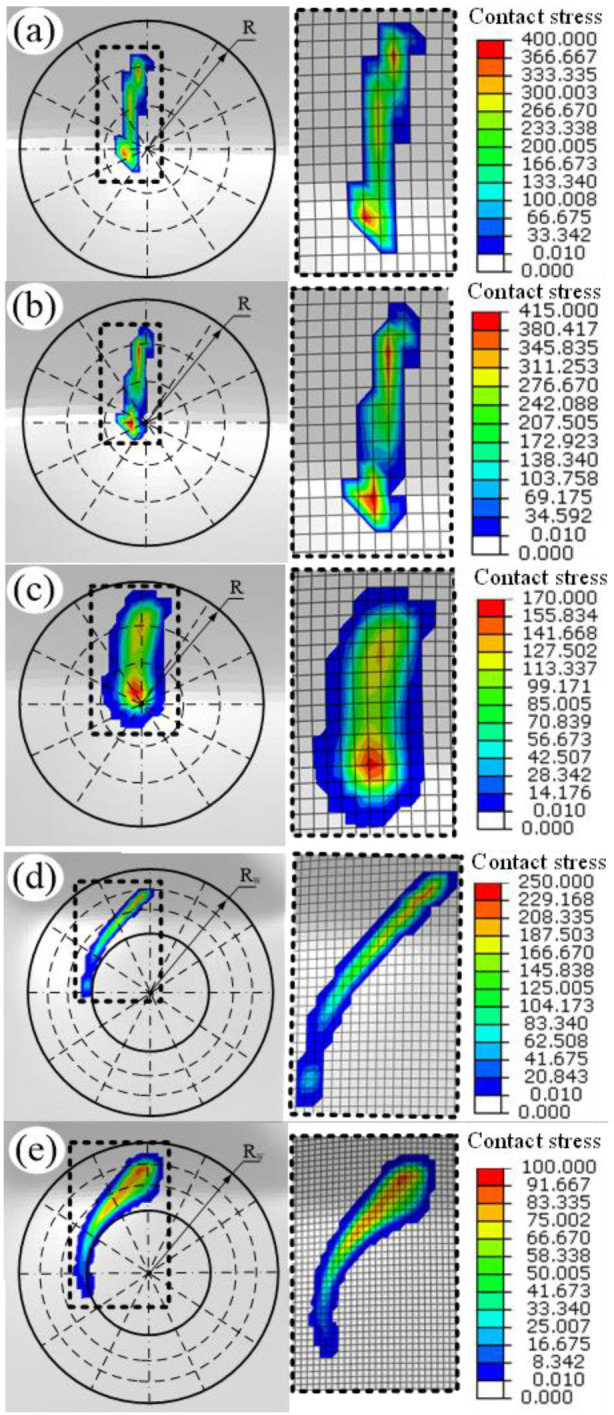
The contacting topography of different forming tools resulted from simulation: (**a**) MFB; (**b**) MRB; (**c**) AFB; (**d**) MRW; (**e**) ARW.

Figure 7 presents the contact maps of different forming tools with sheet obtained through numerical simulation. The result reveals that ball tools with different materials and motion characteristics exhibit identical contact geometries. Due to significant elastic deformation occurring in the acetal tool during the forming process, its contact area is substantially larger than that of metal tools.

**Figure 8:**
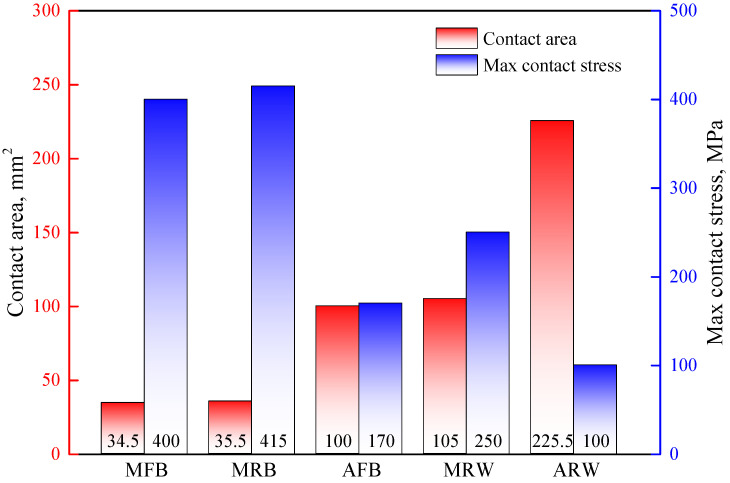
Contacting results using various forming tool: contacting area and contacting stress.

Figure 8 presents a comparative analysis of contact area and contact stress distributions, where the contact area was quantified by calculating the number of contact mesh elements. The metallic ball tool (MFB) exhibited a contact stress of 400 MPa with a contact area of 34.5 mm^2^, while the metallic rolling ball (MRB) showed comparable values of 415 MPa and 35.5 mm^2^ respectively. It should be noted that the reported contact stress values represent the localized interfacial pressure between the tool and sheet, not the bulk von Mises stress within the material. Although these values exceed the uniaxial tensile strength of copper (221.2 MPa), no fracture occurred experimentally or in the simulation. This is because the material is under a multi-axial stress state with significant compressive normal stress at the contact zone, which increases hydrostatic pressure and enhances the forming limit. When using Acetal material, the contact area significantly increased to 100 mm^2^ (189.9% increase versus MFB) while the contact stress reduced to 170 MPa (57.5% reduction). For the larger tool sizes with wheel type, the MRW configuration demonstrated a contact area of 105 mm^2^ (204.4% increase) with 250 MPa stress (37.5% reduction), and the ARW tool achieved the most substantial improvement with 225.5 mm^2^ contact area (553.6% increase) and 100 MPa stress (75.0% reduction). In summary, the deformable acetal tool effectively increases contact area while reducing contact stress during forming, thereby preventing sheet material wear. Furthermore, the proposed wheel rolling tool design demonstrates additional stress reduction capabilities.

### 3.3. Forming Force and Geometrical Outline

**Figure 9:**
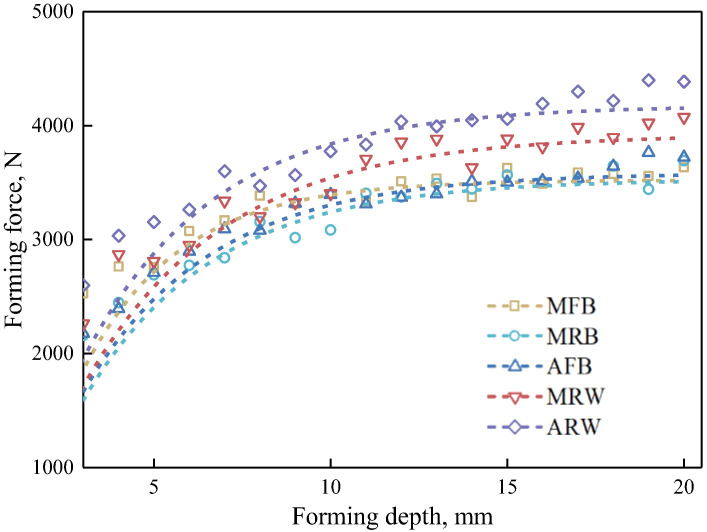
Experimental force history during forming process with various forming tools.

Figure 9 illustrates the variation in resultant forming force with step depth for different tool configurations. The forming force initially increases with step depth before stabilizing, following the fitted relationship:

(2) $$F=F_{0}+Q(1-exp(-q\times z))$$

where $F_{0}$, Q, and *q* are fitting parameters, z is forming depth, with Q representing the peak forming force. The forming force parameters of different tools are listed in Table 2. The peak forming forces for ball tools (3376.61 N for MFB, 3301.81 N for MRB, and 3280.15 N for AFB) show minimal variation, indicating that tool material has limited influence on forming force in ball configurations. In contrast, wheel-type tools exhibit higher forming forces due to their larger contact areas, with the MRW and ARW tools reaching 3616.33 N (7.1% increase) and 3762.90 N (11.4% increase), respectively, compared to the metal ball tool. Notably, the Acetal wheel tool (ARW) generates a higher forming force than its metal counterpart (MRW), suggesting that the increased contact area in wheel-type tools dominates over the stress-reducing effect of acetal material.

**Figure 10:**
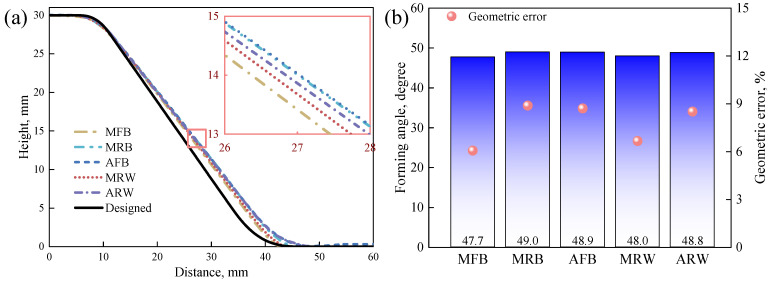
(**a**) Experimental cross-sectional profile and (**b**) geometric errors of parts formed by different forming tools.

Figure 10 presents a comparison of formed-part profiles across different tool configurations, with all experimental profiles showing measurable deviation from the theoretical contour. Detailed examination through the inset window reveals that among metal tools, the MFB configuration produces profiles most closely matching the theoretical contour, while the MRW tool shows slightly greater deviation. Quantitative assessment using angular deviation ($\alpha_{T}$/α, where the design angle *α* = 45°) demonstrates that metal tools achieve superior geometric accuracy. The MFB tool yields a formed angle of 47.7° (6.1% error), closely followed by MRW at 48.0° (6.7% error). The MRB configuration shows marginally greater deviation at 48.9° (8.9% error). Acetal-based tools show lightly increased deviation due to material deformation of tool during forming. The AFB tool produces a formed angle of 48.9° (8.7% error), with the ARW configuration is at 48.8° (8.5% error). These results represent only a 2.4~2.6% increase in error relative to the MFB benchmark, confirming that acetal’s deformation characteristics have controllable impact on geometric precision.

## 4. Discussion

### 4.1. The Influence of Tool Material

**Figure 11:**
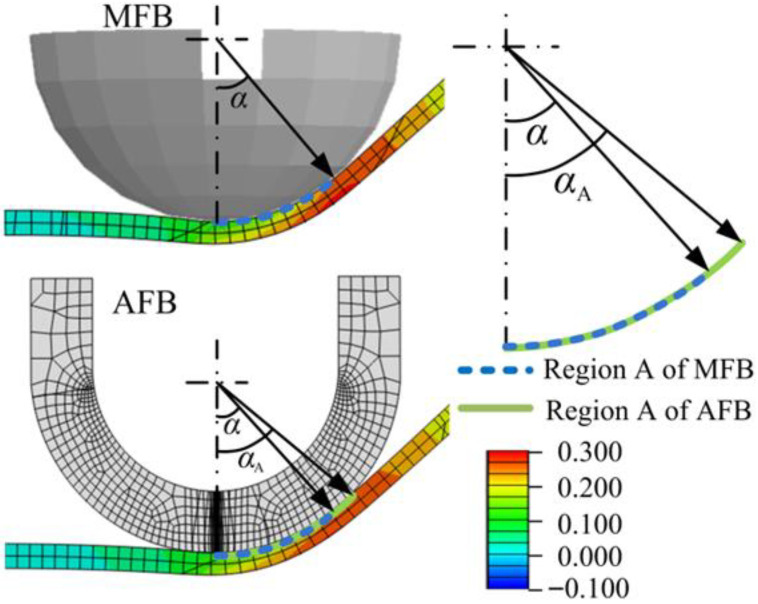
The contacting region using metal and acetal ball tool (MFB and AFB) in simulation results.

**Figure 12:**
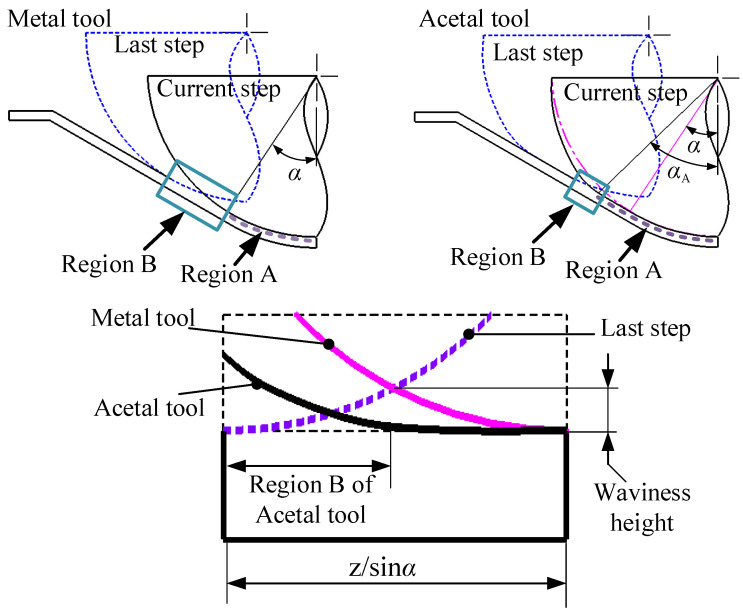
The material influence of forming tools on tool-contacting (region A) and waviness formation (region B) region using metal and acetal ball tools (MFB and AFB).

The significantly lower strength of acetal materials compared to metals leads to elastic deformation of acetal tools during forming processes, resulting in increased contact area as illustrated in Figure 11. As demonstrated in Figure 12, while surface waviness in incremental forming originates from bending deformation in non-contact regions (Region A) [3], the compliant nature of acetal tools expands the tangential material contact zone (Region B). This deformation mechanism increases the contact angle from 45° (design angle α) to 50.7° (contacting angle of acetal tool $\alpha_{A}$) as exported from simulation results, effectively reducing or eliminating Region B dimensions. Consequently, the surface-waviness topography is suppressed, leading to remarkable improvement in part-surface quality.

For identical tool geometries, the enhanced contact area of acetal tools effectively reduces contact stress by 57.5% (ball tool) and 60.0% (wheel-type tool), respectively, significantly decreasing tool wear. This stress reduction stems from the improved pressure distribution across the enlarged contact zone. Furthermore, as the acetal material strength is substantially lower than that of the sheet metal, wear predominantly occurs on the tool surface rather than the workpiece. This protective mechanism ensures superior surface finish of the formed parts while maintaining geometrical accuracy within 2.5% variation.

In summary, the acetal tool demonstrates two key advantages in incremental forming processes: its elastic deformation behavior significantly increases the contact area, effectively suppressing the characteristic waviness formation in incremental forming; and the corresponding reduction in contact stress (by 57.5~60.0%) minimizes surface wear damage. While introducing a marginal 2.4~2.6% increase in geometric deviation, this trade-off remains well within acceptable limits for most industrial applications. These findings collectively demonstrate the strong potential of acetal tools for precision forming applications where surface quality is paramount.

### 4.2. The Influence of Tool Structure

**Figure 13:**
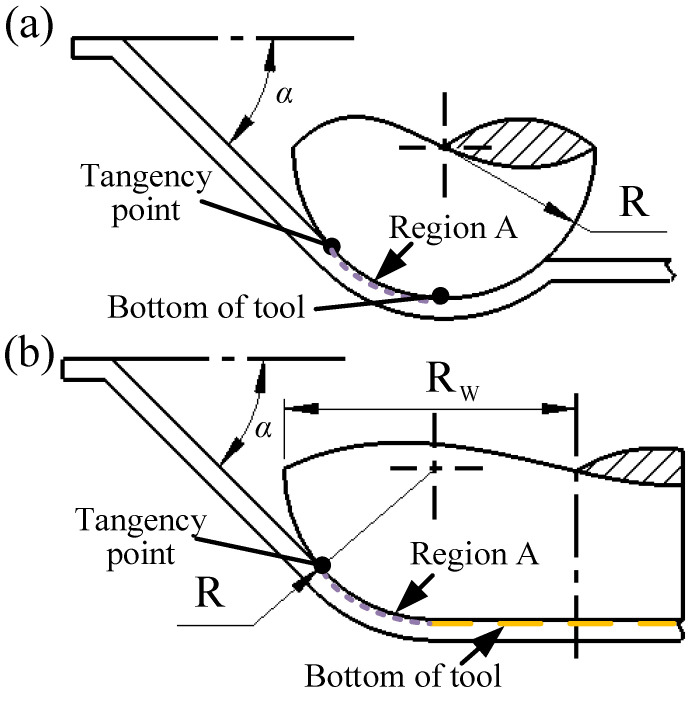
The cross-section illustration of (**a**) ball and (**b**) wheel-type tools.

Figure 13 demonstrates that ball and wheel-type metallic tools exhibit identical contact regions (Region A) with the sheet material, producing similar waviness patterns. The rotation of metallic ball tools does not significantly improve surface quality because the ball geometry cannot maintain stationary contact points with the sheet, resulting in persistent sliding friction conditions comparable to fixed tools [23,24]. Additionally, the contact-stress distribution remains unchanged regardless of rotation, further limiting any potential improvement. In contrast, while wheel-type metallic tools also experience sliding friction and increased forming forces, their larger radial dimensions expand the contact area, reducing contact stress by 37.5% and thereby improving surface quality.

**Figure 14:**
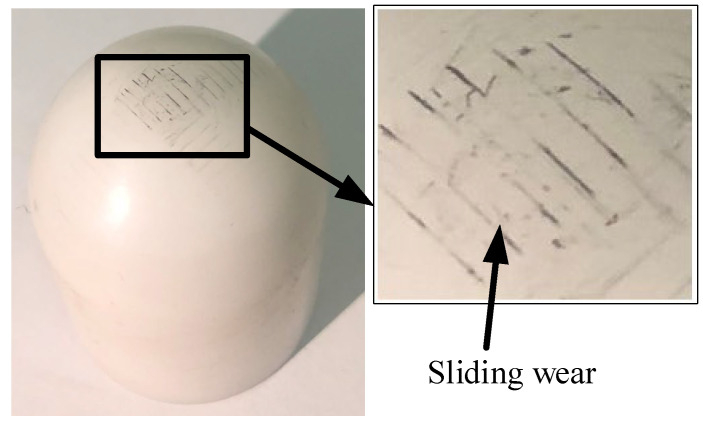
The surface topography of acetal ball tool after ISF process.

Similar to metallic tools, the wheel-type acetal tool demonstrates a 41.2% reduction in contact stress compared to its spherical counterpart. As tool wear in acetal tools predominantly occurs at the contact surface, this stress reduction directly mitigates wear progression. The AFB exhibits severe surface degradation after forming (as shown in Figure 14), while the ARW maintains its original surface topography. Notably, geometric deviations in formed parts remain comparable between wheel-type and ball tools for both metallic and acetal variants. These results demonstrate that wheel-type tools, particularly the ARW configuration, exhibit strong potential for industrial implementation due to their dual capability of significantly reducing contact stresses while maintaining dimensional accuracy.

## 5. Conclusions

Surface quality has remained the critical challenge hindering the widespread adoption of incremental sheet forming (ISF) technology. To address this issue, this study systematically investigates various tool materials and structural designs through integrated experimental and numerical approaches. The key findings are summarized as follows:

1. The newly developed acetal rolling-wheel tool demonstrates remarkable improvements in surface quality, showing great potential for industrial applications. Compared with conventional metal tools, it achieves a 32.0% enhancement in surface finish while maintaining geometric accuracy with only a marginal 2.4% increase in dimensional deviation.
2. The acetal tool improves surface quality through two synergistic effects: increased tangential contact area that effectively eliminates waviness formation, and reduced contact stress (by 57.5% for ball tools and 60.0% for wheel-type tools) that minimizes surface friction damage.
3. The wheel-type tool design provides additional benefits through its enlarged radial dimensions. The metallic wheel tool reduces contact stress by 37.5% and improves surface quality by 21.1% compared to the ball tool. Furthermore, the acetal wheel tool achieves a 41.2% stress reduction while completely preventing tool surface wear, substantially enhancing its practical utility in manufacturing applications.

Future work will extend to various materials (including aluminum alloys and steels) and complex geometries, while continuing to explore alternative tool materials beyond acetal to enhance wear resistance for broader industrial application.

## Figures and Tables

**Table 1:** Forming parameters during ISF process.

Parameter	Step Depth	Feed Rate	Tool Radius
Value	1.0 mm	3000 mm/min	10 mm

**Table 2:** The forming force parameters of different forming tools.

Coefficient	MFB	MRB	AFB	ARW	MRW
$F_{0}$	144.57	225.03	302.03	304.67	413.25
Q	3376.61	3301.81	3280.15	3616.33	3762.90
q	0.36	0.27	0.28	0.25	0.27

## Data Availability

The original contributions presented in this study are included in the article/Supplementary Material. Further inquiries can be directed to the corresponding authors.

## Supplementary Materials

The following supporting information can be downloaded at: https://www.mdpi.com/article/10.3390/ma19040826/s1, Figure S1 compares the simulated and experimental forming forces along the z-axis during the process for all five tool configurations. The results show that the simulation effectively captures the trend of the forming force. In the steady-state stage (15–25 s), the average force errors for MFB, MRB, AFB, MRW, and ARW are 14.8%, 12.8%, 13.8%, 13.0%, and 4.4%, respectively. All errors are below 15%, confirming the reliability of the simulation results.
