# Supplementary material for: Novel Acetal Rotating Tool Improving Surface Quality in Incremental Sheet Forming
Source: Materials (Basel). 2026 Feb 23;19(4):826. doi: 10.3390/ma19040826 (PMC12941510; doi:10.3390/ma19040826)
Supplement: Supplementary file 1 [file materials-19-00826-s001.zip › materials-4116446-supplementary.pdf]

Supplementary Materials:

Figure S1 compares the simulated and experimental forming forces along the z-axis during the process for all five tool configurations. The results show that the simulation effectively captures the trend of the forming force. In the steady-state stage (15–25 s), the average force errors for MFB, MRB, AFB, MRW, and ARW are 14.8%, 12.8%, 13.8%, 13.0%, and 4.4%, respectively. All errors are below 15%, confirming the reliability of the simulation results.

(a)

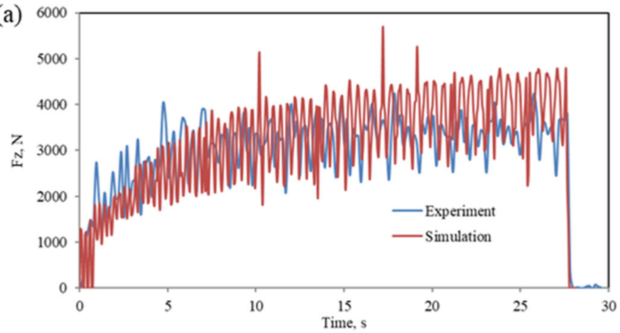

(b)

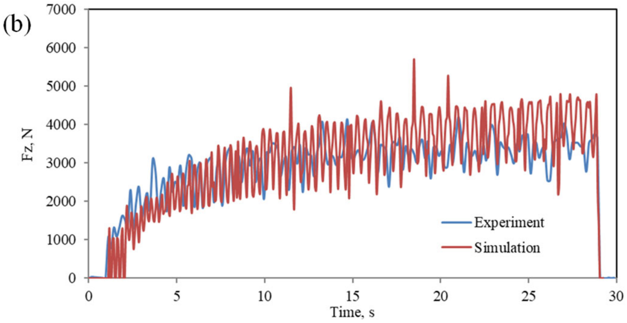

(c)

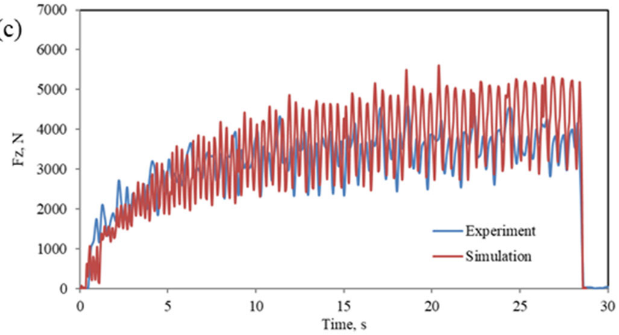

(d)

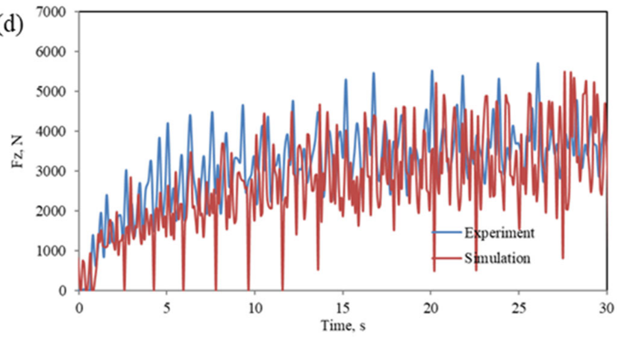

(e)

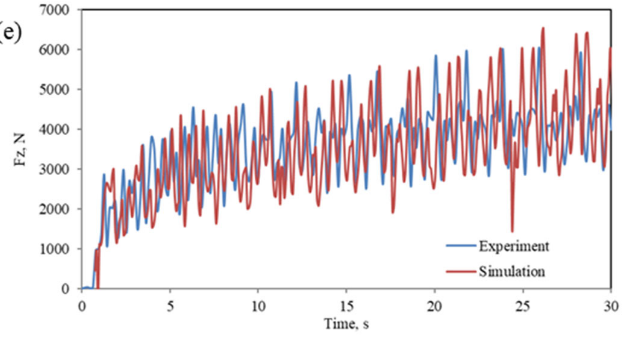

Fig.S1 Force histories comparison between experiment and simulation using various forming tools: (a) MFB; (b) MRB; (c) AFB; (d) MRW; (e)ARW
